# Lipopolysaccharide regulated protein expression is only partly impaired in monocytes from patients with type I diabetes

**DOI:** 10.1186/1475-2840-5-5

**Published:** 2006-03-27

**Authors:** Gabriele Wehrwein, Markus Neumeier, Andreas Schäffler, Andrea Kopp, Johanna Weigert, Sabine Abke, Jürgen Schölmerich, Christa Buechler

**Affiliations:** 1 Department of Internal Medicine I, University of Regensburg, D-93042 Regensburg, Germany

## Abstract

**Background:**

Monocytes play an important role in innate immunity and atherosclerosis. A disturbed secretion of cytokines in lipopolysaccharide (LPS) activated monocytes from type 1 diabetes (T1D) patients has been described and may contribute to the impaired inflammatory response in these individuals. In the present study the influence of LPS on five different proteins with a function in immunity and atherosclerosis was analyzed in monocytes from controls and T1D patients.

**Methods:**

Monocytes were isolated from controls and T1D patients and the LPS-stimulated increase of IL-6, CXCL8, monocyte chemotactic protein 1 (CCL2, MCP-1) and superoxide dismutase (SOD 2), as well as the LPS-mediated decrease of apolipoprotein E (Apo E) in primary human monocytes from controls and T1D patients was determined.

**Results:**

CCL2 and IL-6 secretion in response to LPS was found significantly reduced in monocytes from T1D patients when compared to controls whereas basal CCL2 release was similar in control and T1D cells. In contrast, CXCL8 and apolipoprotein E secretion and SOD 2 expression upon LPS stimulation is similar from T1D and control monocytes.

**Conclusion:**

These data indicate that LPS-mediated protein expression is only partly disturbed in monocytes from T1D patients. Reduced secretion of IL-6 and CCL2 in activated monocytes of these patients may contribute to an impaired inflammatory response and vascular disease.

## Background

Type 1 diabetes mellitus (T1D) is a complex disease with genetic and environmental factors involved in its etiology [1,2]. T1D carries a substantial risk of morbidity and early mortality due to its complications, which affect the macro- and microvasculature [3]. An impaired production of cytokines by monocytes and macrophages of these patients has been associated with the pathophysiology of T1D. Monocytes play an important role in the innate immune response and vascular complications and an abnormal cytokine release may contribute to premature atherosclerosis and reduced immune function in T1D patients [4,5]. Several studies demonstrate a disturbed response of T1D monocytes to endotoxin shown by an altered cytokine secretion [6]. In one study purified monocytes of T1D patients treated with LPS had an elevated release of IL-6 and IL-10 whereas TNF was not altered [7]. Other reports, however, describe reduced IL-6 and IL-1 levels [8] or diminished IL-6 and IL-10 release of endotoxin activated T1D monocytes [9]. The non-obese diabetic (NOD) mouse is a widely used animal model to study T1D and peritoneal macrophages activated with LPS have reduced IL-1 and TNF secretion whereas IL-6 and IL-10 were not altered [10]. Leukocytes of these mice have a severely impaired migration towards sites of inflammation that might be partly explained by a disturbed cytokine profile in these animals [11].

Cytokines and chemokines are the main group of genes induced by LPS [12]. Chemokines are key signal molecules that attract cells of the immune system to the site of inflammation and also have a prominent role in the formation of early atherosclerotic lesions. The most thoroughly characterized CC chemokine is CCL2 (MCP-1) and several studies provide evidence that CCL2 is the main chemokine involved in the recruitment of monocytes from blood into early lesions. However, NOD exsudate macrophages and monocytes from T1D patients display a severely reduced migration towards CCL2 [11,13]. CXCL8 (IL-8), a CXC chemokine is also induced by LPS, stimulates the adhesion of monocytes to endothelial cells and has also been linked to the development of early atherosclerosis [14].

Manganese superoxid dismutase (SOD 2) is also upregulated by LPS and protects the cell against damage by superoxide radicals [15]. Decreased activity of this enzyme has been associated with the pathogenesis of T1D [16] and an enhanced release of superoxide has been reported in neutrophils from poorly controlled T1D patients [17].

Apolipoprotein E (Apo E) inhibits local inflammatory responses [18] and enhances the clearance of cellular lipids [19]. Apo E release is suppressed in LPS-activated monocytes and this may contribute to an enhanced secretion of type I cytokines and lipid accumulation in activated macrophages [20].

LPS may be linked to vascular disease and low levels of circulating endotoxin in men and rabbits promote the development of atherosclerosis [21,22]. Deficiency of the LPS receptor TLR4 or MyD88 involved in LPS-signaling decrease plaque size and macrophage infiltration [23,24]. These recent findings indicate that an altered inflammatory response of T1D monocytes may not only affect innate immunity but in addition premature cardiovascular disease in these patients. Therefore we investigated the response of T1D monocytes and control cells to LPS as an proinflammatory and proatherogenic mediator. The secretion of the multifunctional cytokine IL-6 [25] and the chemokines CCL2 and CXCL8, that attract cells of the immune system like monocytes and are released by these cells, was investigated [26]. The abundance of Apo E, which mediates lipid efflux from monocytes and exerts immunsuppressive effects [27] and the expression of the radical scavenging enzyme SOD 2 were determined [28].

## Methods

### Patients and controls

Monocytes were purified from the blood of 10 female controls and 10 female T1D patients. The median age of the controls was 24 years (range 24 – 43) and of the patients 36.5 years (range 18 – 46). The mean body mass index (BMI) of controls was 20.6 kg/m^2 ^(range 17.5 – 22.3) and of the T1D patients 22.3 kg/m^2 ^(range 19.5 – 31). Only patients with a known history of T1D and an established therapy with intensive insulin treatment were recruited for the study. The median duration of diabetes was 13.5 years (range 7 – 34). The mean HbA1c was 7.2 % (range 5.7 – 9.5). Patients had no infectious disease within two weeks before blood was drawn. C-reactive protein was determined by an ELISA from Anogen (Ontario, Canada) in the plasma and was 1.94 mg/l (range 0.5 – 5) in controls and 1.1 mg/l (range 0.7 – 1.6) in T1D. All women gave informed consent and the study was approved by the local Medical Ethical Committee.

### Reagents

Macrophage SFM medium was from Gibco BRL (Karlsruhe, Germany). Recombinant M-CSF and CXCL8 ELISA were from R&D Systems (Wiesbaden-Nordenstadt, Germany), IL-6 ELISA was from Pierce Biotechnology (Rockford, Illinois), and CCL2 ELISA was obtained from Amersham Biosciences (Freiburg, Germany). E. coli derived recombinant human proteins were used as standard for the IL-6, CXCL8 and CCL2 ELISAs. SOD 2 antibody was from Abcam (Cambridge, UK) and Apo E antibody was from Chemicon (Hampshire, U.K.). Vacutainer CPT were from Becton Dickinson (Franklin Lakes, NJ). LPS, *E. coli *serotype 055:B5 was ordered from Sigma Chemicals (Deisenhofen, Germany). Apo E ELISA was from MoBiTec (Goettingen, Germany) and the calibrator is derived from human serum.

### Isolation and culture of primary blood monocytes

Peripheral blood leukocytes were isolated from 16 ml of whole blood by Vacutainer CPT and monocytes were further purified by magnetic separation with CD14 beads (Miltenyi Biotec, Bergisch Gladbach, Germany). Purity of the isolated monocytes was determined by flow cytometric analysis and was more than 98%. 500,000 monocytes were cultivated in 500 μl macrophage SFM medium in 24 wells plates with 50 ng/ml M-CSF for 24 h. Subsequently the medium was replaced. Monocytes were either cultivated in 500 μl macrophage SFM medium with M-CSF or in the identical media supplemented with 1 μg/ml LPS. Supernatants and cells were collected 24 h later and used for ELISA or immunoblotting.

### SDS-PAGE and immunoblotting

The cells were solubilized in RIPA buffer. 8 μg of protein were separated by SDS-polyacrylamide gel electrophoresis and were transferred to PVDF membranes (Bio-Rad, Germany). Incubations with antibodies were performed in 1% BSA in PBS, 0.1% Tween overnight. Detection of the immune complexes was carried out with the ECL Western blot detection system (Amersham Pharmacia, Deisenhofen, Germany).

### Statistics

Data are represented as Box Plots indicating the median, the upper and lower quartile, the largest and the lowest value in the data set. In the manuscript data are given as median value and the range of the values. Statistical differences were analyzed by two tailed Mann-Whitney U Test and a value of *P *< 0.05 was regarded as statistically significant.

## Results

### LPS-induced IL-6 secretion in monocytes from controls and T1D patients

**Figure 1:**
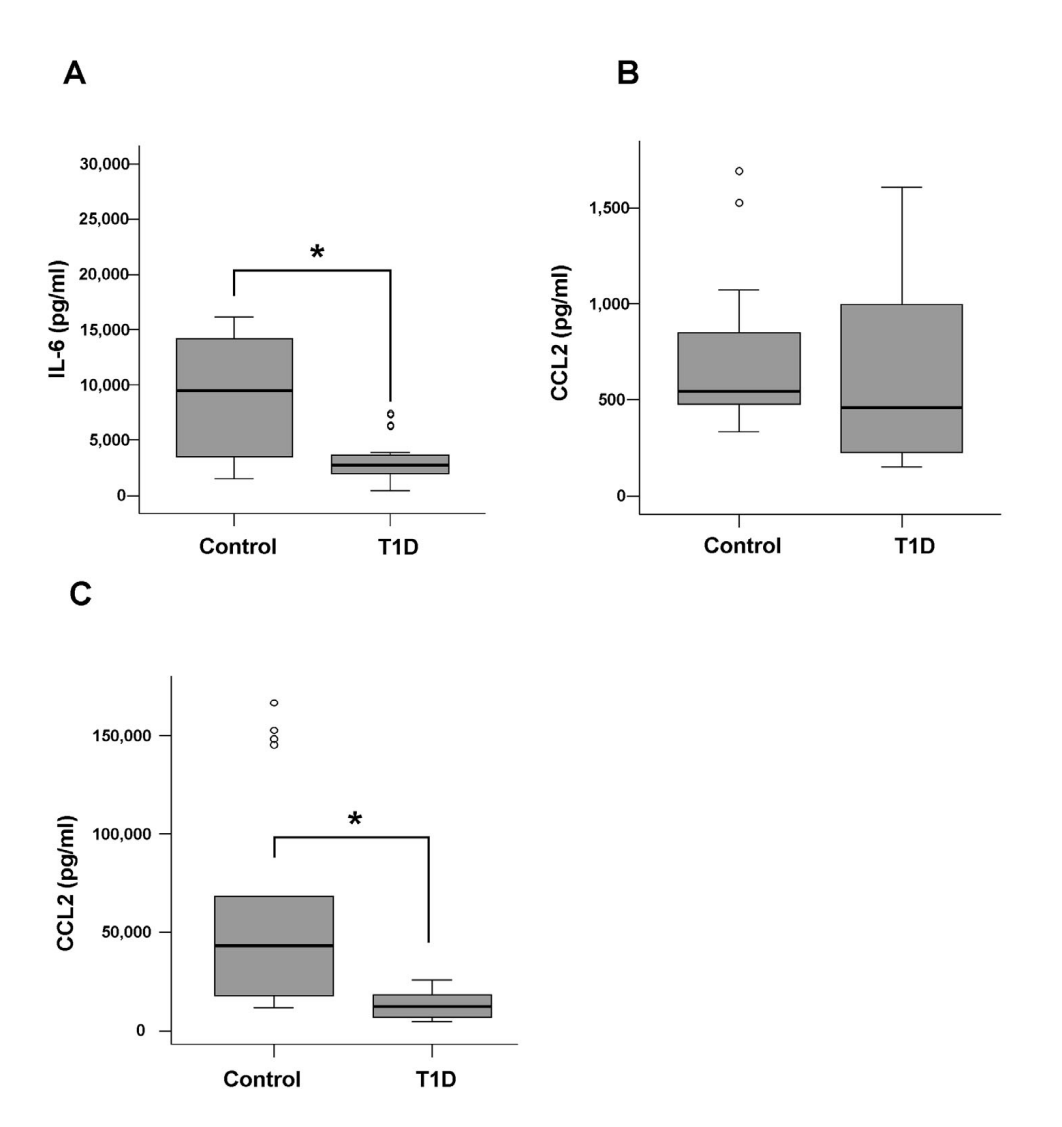
**LPS-stimulated IL-6 and CCL2 secretion of T1D and control monocytes**. Monocytes from 10 controls and 10 T1D patients were cultivated as described in the text. IL-6 (A) and CCL2 (C) were determined in the supernatant of cells treated with 1 μg/ml LPS for 24 h. CCL2 was also determined in unstimulated monocytes (B). Outliers are indicated by the dots.

IL-6 was determined in the supernatants of monocytes treated with LPS or cultured in medium alone by ELISA. IL-6 was not detected in cells from controls and monocytes from T1D patients not treated with LPS indicating that only low amounts of IL-6 are released (not shown). This demonstrates that non-activated monocytes were used for the study. IL-6 was markedly elevated in the supernatants of LPS incubated cells. The median of LPS induced secretion in monocytes from controls was 9,469 pg/ml (range 1,503–16,000) and in monocytes isolated from T1D patients 2,742 pg/ml (range 775–7,431) (Figure 1A) indicating a significant reduced IL-6 release in monocytes from T1D patients (p = 0.001).

### Basal and LPS-induced CCL2 secretion in monocytes from controls and T1D patients

The supernatants described above were also used to measure CCL2. CCL2 is constitutively secreted by non-stimulated monocytes and was 543 pg/ml (range 369–1,693) in controls and 461 pg/ml (range 202–1,604) in monocytes from T1D patients (Figure 1B) and therefore is similar in unstimulated monocytes isolated from controls or T1D patients. In LPS-treated cells, CCL2 secretion is strongly induced. CCL2 secreted from control monocytes was 39,882 pg/ml (range 9,698–74,597) and from T1D 9,961 pg/ml (range 4,509–22,980). LPS-induced release of CCL2 is lower in monocytes from T1D patients when compared to controls and this difference is statistically significant (p = 0.00014) (Figure 1C).

### Basal and LPS-induced CXCL8 secretion in monocytes from controls and T1D patients

**Figure 2:**
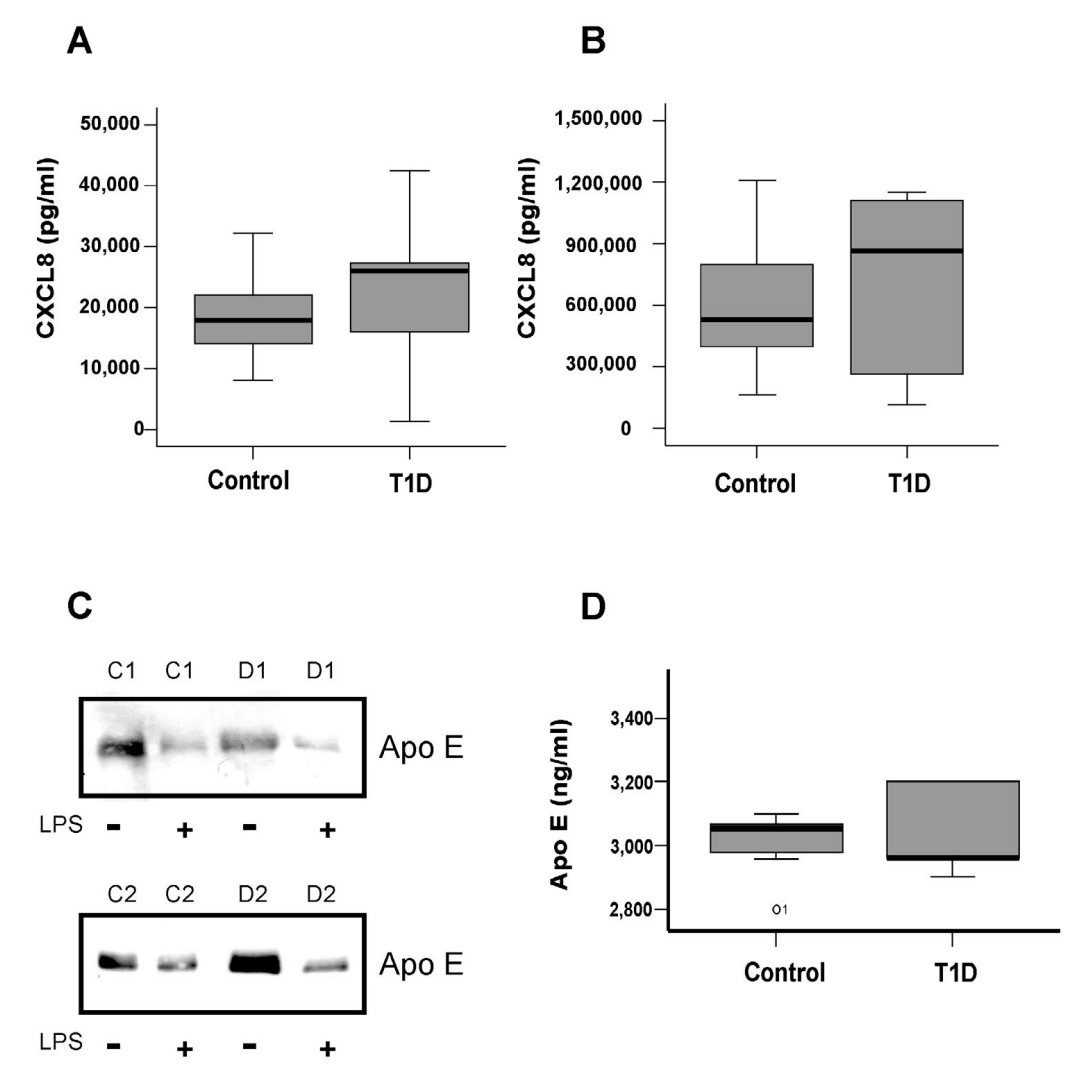
**LPS-mediated CXCL8 and Apo E in T1D and control monocytes**. Monocytes from controls and T1D patients were cultivated with or without LPS for 24 h. CXCL8 was determined in non-activated monocytes of controls and T1D patients (A) and LPS-activated cells (B). Intracellular Apo E was analyzed by immunoblot and the result for monocytes from control 1 and 2 (C1, C2) and T1D patient 1 and 2 (D1, D2) is shown (C). Apo E was determined in the supernatants of 10 control and 10 T1D monocytes treated with 1 μg/ml LPS for 24 h. Outliers are indicated by the dots (D).

Furthermore CXCL8 was determined in the supernatants by ELISA. CXCL8 is already expressed in non-stimulated monocytes and was 19,252 pg/ml (range 8,002–40,221) in controls and 24,126 (1,302–42,504) in monocytes from T1D patients indicating a similar release of this chemokine (p = 0.47) (Figure 2A). CXCL8 in LPS-activated monocytes from controls was 659,000 pg/ml (range 257,000 – 1,710,000) and 890,000 pg/ml (range 185,000–1,150,000) in T1D monocytes (p = 0.26) (Figure 2B).

### Apo E release in LPS-stimulated cells

Apo E secretion is suppressed by LPS and this was confirmed in our study by immunoblot using cell lysates from control and T1D monocytes (Figure 2C). Apo E release was measured by ELISA in LPS-activated monocytes and was 3,017 ng/ml (range 2,800–3,100) from controls and 2,961 ng/ml (range 2,957–3,200) from T1D monocytes and therefore was similar in LPS-activated monocytes from controls and T1D patients (Figure 2D).

### LPS-induced SOD 2 in monocytes from controls and T1D patients

**Figure 3:**
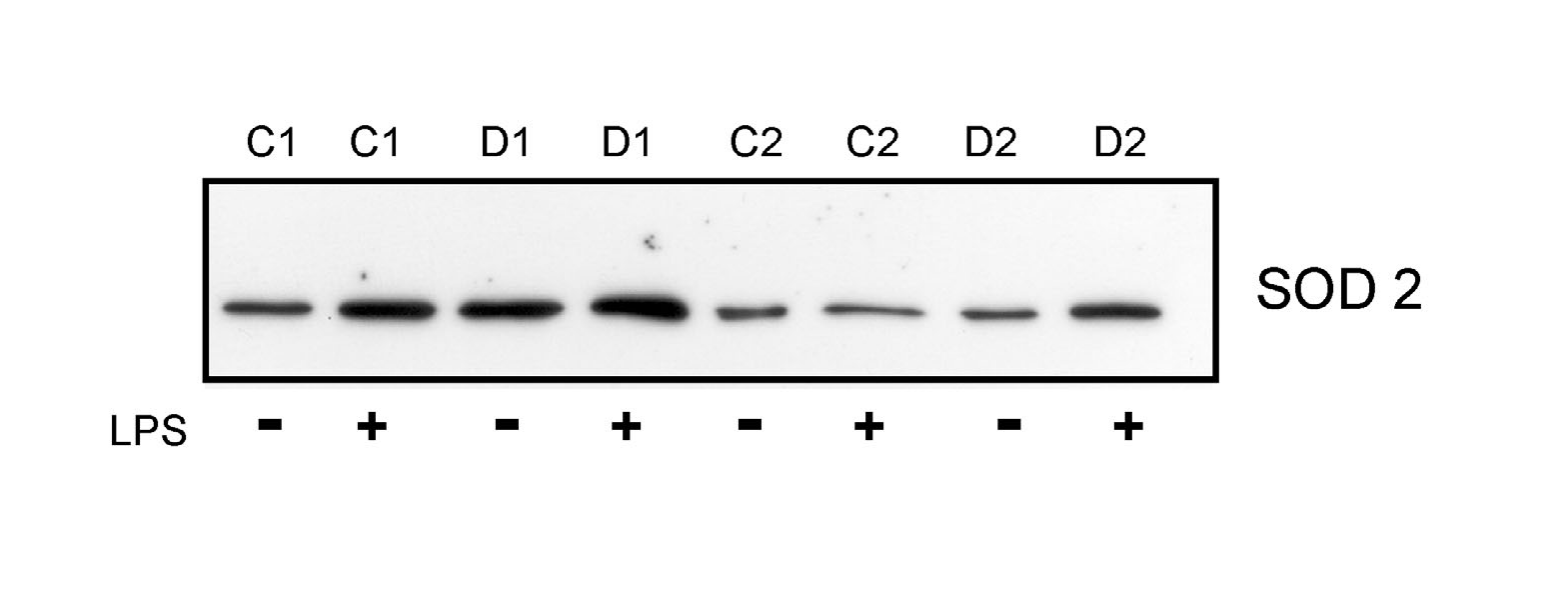
**SOD 2 in LPS-stimulated T1D and control monocytes**. Monocytes from four different controls (C1 to C4) and four different T1D patients (D1 to D4) were cultivated with or without LPS for 24 h. SOD 2 was determined by immunoblot and a representative immunoblot from control 1 and 2 (C1, C2) and T1D patient 1 and 2 (D1, D2) is shown.

SOD 2 was analyzed by immunoblot in monocytes isolated from six controls and six T1D patients. With the exception of cells isolated from one control individual (C2 in figure 3), the expression of SOD 2 was induced by LPS. A representative immunoblot is shown in Figure 3. Quantification of the immunoblots using the OptiQuant software revealed that SOD 2 is 2.0 ± 1.4 fold induced in controls and 3.2 + 1.8 fold induced in T1D cells indicating a similar upregulation of SOD 2 by LPS in control and patients cells.

## Discussion

When compared to monocytes isolated from controls, T1D cells show a significantly lower secretion of IL-6 and CCL2 upon LPS-stimulation. The reduced IL-6 release from activated monocytes is a confirmatory finding and has already been published by two studies ([8,9]. In contrast, Spatz et al. describe an elevated IL-6 in T1D cells [7]. The reason for this discrepancy is so far unclear but neither duration of diabetes nor gender of the patients may explain this different findings. Increased serum levels of IL-6 have been found in patients with stroke or type 2 diabetes mellitus and IL-6 is suggested to increase the risk of coronary artery disease [29,30]. This may indicate that reduced IL-6 release of LPS-activated T1D monocytes is protective in the development of atherosclerosis. However, impaired IL-6 secretion may contribute to an ineffective innate immune response and higher incidence and duration of infections [31] and this may secondary promote atherogenesis.

Diminished secretion of CCL2 by LPS-stimulated T1D cells has not been described so far. This chemokine is a potent agonist for monocytes and T-cells. Levels of CCL2 are increased early in the course of plaque formation and lead to increased monocyte migration into the atherosclerotic lesion [26]. Monocytes migrate into the intima due to a CCL2 concentration gradient that is formed by endothelial cells and monocytes [32]. Lower expression of CCL2 by activated T1D cells may increase this gradient and enhance migration of monocytes to the endothelium. Furthermore CCL2 is decreased in the serum T1D patients [33] and diminished secretion of CCL2 by activated monocytes may contribute to reduced serum levels. Depletion of CCL2 was demonstrated to alter the expression of at least nine additional genes [34] showing that the chemokine network is highly sensitive to any alterations. The chemokine CXCL8 also activates monocytes and may recruit these cells to the endothelium. CXCL8 was so far not analyzed in T1D monocytes but was found similar abundant in the serum of T1D patients and controls [35]. This chemokine is secreted by various cells and here we describe that CXCL8 release is similar in non-stimulated and LPS-activated monocytes from T1D patients when compared to controls.

SOD 2 converts superoxide to oxygen plus hydrogen peroxide and serves as the primary defense against mitochondrial superoxide [15]. Enhanced oxidative stress may promote inflammation and atherosclerosis [36]. Furthermore, superoxides potentiate LPS-stimulated production of IL-6 [37]. However, LPS-induced SOD 2 expression was similar in monocytes of T1D cells and controls indicating that an altered regulation of SOD 2, that may be associated with elevated or reduced superoxide release, does not explain altered inflammatory response in T1D nor premature cardiovascular disease.

Apo E is important for lipid efflux of monocytic cells and Apo E deficiency in mice is associated with atherosclerosis. Loss of Apo E results in an impaired clearance of apoptotic cell remnants by phagocytes and leads to systemic proinflammatory conditions [27]. Apo E synthesis and secretion is suppressed by LPS in monocytes [20,38] and was found similar abundant in endotoxin treated monocytes from controls and T1D patients. Apo E protects monocytes from lipid accumulation [19] and furthermore reduces the release of type I inflammatory cytokines like IL-6 in LPS-treated mice [18]. Because Apo E release is similar in LPS-activated monocytes from controls and T1D patients, an altered Apo E secretion in T1D cells can be ruled out to explain reduced IL-6 release. Deficiency of macrophage Apo E is strongly associated with atherogenesis in mice [19] but may not be a factor in premature cardiovascular disease in T1D.

These data indicate that LPS-activated signal transduction pathways are only partly impaired in T1D monocytes. Though it is clear that the expression of the proteins investigated in the present study is regulated by LPS, different intracellular signal transduction pathways are most likely involved. Whereas induction of IL-6 and CCL2 depends on the mitogen-activated protein kinases ERK1/2 and p38 [39], upregulation of CXCL8 is mediated by ERK1/2 but not p38 [40] and SOD 2 is upregulated independent of MAPK [15]. LPS-mediated reduction of Apo E may be facilitated by an autocrine, TNF-dependent mechanism [20]. However, LPS-signaling is highly complex and various proteins and transcription factors are involved until an altered protein expression is achieved [38]. Therefore additional studies are needed to identify the pathways that are affected in T1D monocytes.

The T1D patients in our study are relatively young with so far no secondary complications of T1D. The median HbA1c value of 7.2% indicates that the diabetes is well controlled. Therefore, the observed diminished inflammatory response of the T1D monocytes is most likely not a secondary effect of T1D but may be an intrinsic property of the cells.

## Conclusion

Taken together, the present study demonstrates a diminshed release of IL-6 and CCL2 from LPS activated T1D monocytes indicating an impaired primary immune response that subsequently promotes atherosclerosis in these patients.

## Abbreviations

Apolipoprotein E (Apo E), extracellular signal-regulated protein kinase (ERK), interleukin-6 (IL-6), interleukin-8 (IL-8; CXCL8), lipopolysaccharide (LPS), macrophage colony stimulating factor (M-CSF), mitogen-activated protein kinase (MAPK), monocyte chemotactic protein 1 (CCL2, MCP-1), myeloid differentiation primary response gene (MyD88), non-obese diabetic (NOD), superoxide dismutase (SOD 2), toll-like receptor 4 (TLR4), tumor necrosis factor (TNF), type 1 diabetes (T1D).

## Competing interests

The author(s) declare that they have no competing interests.

## Authors' contributions

GW collected the patients and control samples and carried out immunoassays; MN carried out immunoassays; AS participated in the design of the study; AK, JW and SA carried out immunoassays, JS is the head of the department and provided the required resources for research and was involved in fruitful discussions and preparation of the manuscript; CB conceived of the study, and participated in its design and coordination.
